# Protective role of pinocembrin in a rat model of intestinal ischemia-reperfusion injury

**DOI:** 10.1590/acb403925

**Published:** 2025-06-06

**Authors:** Osman Bardakçı, Hakim Çelik, İlyas Özardalı, Ali Uzunköy

**Affiliations:** 1 Isparta City Hospital – Department of Surgical Oncology – Isparta, Turkey.; 2 Harran University – Medical Faculty – Department of Physiology – Sanliurfa, Turkey.; 3 Kocatepe University – Medical Faculty – Department of Pathology – Afyon, Turkey.; 4 Harran University – Medical Faculty – Department of Surgical Oncology – Sanliurfa, Turkey.

**Keywords:** Intestines, Ischemia, Reperfusion, Oxidative Stress

## Abstract

**Purpose:**

To determine whether pinocembrin (PC) confers protective effects against experimentally induced intestinal ischemia-reperfusion (I/R) injury in rats.

**Methods:**

Thirty Wistar albino rats were randomly divided into three groups (n = 10 each): sham (underwent laparotomy only); I/R (superior mesenteric artery occlusion for 60 min followed by 60 min reperfusion); and I/R + PC (5 mg/kg PC intraperitoneally before ischemia and again prior to reperfusion). Total antioxidant capacity (TAC), total oxidant status (TOS), and oxidative stress index (OSI) were measured in both plasma and intestinal tissue. Histopathological evaluation was performed using hematoxylin and eosin staining and a modified Chiu scoring system.

**Results:**

Although TAC values did not show significant intergroup differences (*p* > 0.05), TOS and OSI values were significantly lower in the I/R + PC group than in the I/R group (*p* < 0.05). Histologically, the I/R + PC group displayed noticeably reduced mucosal damage compared to the untreated I/R group. These results suggest that PC alleviates oxidative stress and improves histological outcomes in intestinal I/R.

**Conclusion:**

PC exhibits a protective effect against intestinal I/R injury by decreasing oxidative stress and preserving tissue architecture. Further studies are warranted to optimize PC’s dosing, timing, and mechanistic actions for clinical application.

## Introduction

Intestinal ischemia-reperfusion (I/R) injury is a severe clinical problem encountered in conditions such as acute mesenteric ischemia, strangulated herni certain shock states and during intestinal transplantation^1^^,^ 2. During ischemia, depletion of adenosine triphosphate (ATP) and the accumulation of reactive oxygen species (ROS) precursors occur^3^^,^ 4. Upon reperfusion, the reintroduction of oxygen paradoxically exacerbates injury by increasing oxidative stress, eliciting strong inflammatory responses, and leading to cell death^5^. Consequently, I/R may disrupt the mucosal barrier, leading to bacterial (endotoxin) translocation and sepsis^6^^–^ 8.

Research on the pathophysiologic mechanisms of intestinal I/R and its resolution is mainly carried out in animal models. Pinocembrin (5,7-dihydroxyflavanone) (PC), a major flavonoid in propolis, possesses antioxidant, anti-inflammatory, antimicrobial, antiapoptotic, and vasorelaxant properties^9^^,^ 10. Many experimental studies have highlighted PC’s beneficial effects in various disease models, including colitis^11^^,^ 12 and cerebral ischemic injury^13^.

Despite growing interest, data on its direct role in preventing or mitigating intestinal I/R-induced injury remain limited. Nonetheless, several studies on ulcerative colitis and other inflammatory bowel disease models have demonstrated the potential of PC in preserving mucosal integrity, reducing cytokine production, and modulating gut microbiota^14^^,^ 15.

Given these findings, we hypothesized that PC might confer protection against the oxidative stress and inflammation inherent to intestinal I/R. The present study aimed to investigate whether PC administration attenuates morphological and biochemical markers of I/R-induced intestinal damage in rats.

## Methods

### Animals

The study was approved by the Harran University Animal Experiments Local Ethics Committee (approval no. 2010/05.07.00/270-40). Thirty male Wistar albino rats (180–240 g) were housed in temperature-controlled conditions (20–22°C), exposed to a 12-h light/dark cycle, and allowed free access to standard rat chow and water. Although the animals were routinely screened for common pathogens, they were not classified as fully specific pathogen-free (SPF).

### Surgical procedures

Rats were randomized into three groups (n = 10 each):

- Sham group: animals underwent midline laparotomy under sterile conditions, but no occlusion of the superior mesenteric artery (SMA) was performed;
- I/R group: after laparotomy, the SMA was clamped with a microvascular bulldog clamp for 60 min to induce ischemia. The clamp was then removed, allowing 60 min of reperfusion;
- I/R + PC group: identical I/R procedures were performed, and PC (5 mg/kg, intraperitoneally) was administered immediately prior to both ischemia and reperfusion.

All procedures were performed under ketamine (87 mg/kg, Ketalar, Pfizer, Turkey) and xylazine HCl (13 mg/kg) anesthesia. At the end of the reperfusion period, tissue samples from the small intestine were harvested for biochemical and histological analyses, and blood was collected by cardiac puncture for plasma assays.

No additional dose of anesthesia was required during the procedure. Intestinal tissues were placed in 10% formaldehyde solution and sent to the pathology laboratory. Paraffin sections were prepared, and 5-micron thick sections were obtained. After deparaffinization, these sections were stained with hematoxylin and eosin stain for histopathological examination. The blood samples obtained from the rats were sanrifuged, and the sera obtained were stored in the biochemistry laboratory at -80°C deep freezer for study. Tissue samples obtained from the same rats were also stored in -80°C deep freezer.

### Biochemical analysis

#### Total antioxidant capacity and total oxidant level

Total antioxidant capacity (TAC) and total oxidant (TOS) levels were measured on a Beckman Coulter AU680 analyzer (Beckman Coulter, Miami, FL, United States of America) using commercial reagents (Rel Assay Diagnostic, Gaziantep, Turkey) based on new automated measurement methods developed by Erel^16^^,^ 17. TAC levels were expressed as mmol Trolox Eq/mg protein. TOS levels were expressed as μmol H2O2 Eq/mg protein^16^^,^ 17.

#### Oxidative stress index

When calculating the oxidative stress index (OSI) of the samples, TAC values are multiplied by 10, and units are equalized with TOS. TAC values were multiplied by 10 to match TOS units, and the oxidative stress index (OSI) was calculated as the ratio of TOS to TAC, expressed in arbitrary units (AU)17. Results were expressed in arbutrary units (AU).

### Histopathological evaluation

Intestinal tissue samples fixed in 10% formalin were embedded in paraffin, sectioned at 5 µm, and stained with hematoxylin and eosin. All histopathological examinations were conducted by a blinded pathologist. The severity of ischemia-reperfusion injury was scored according to the Chiu system, which ranges from 0 (normal) to 5 (severe injury)^18^. Representative sections were photographed, and arrows/markers were used to highlight features such as epithelial lifting, hemorrhage, and edema (Table 1).

**Table 1:** Chiu scoring system.

Score	Histopathologic finding
0	Normal mucosal villi
1	Development of a subepithelial space, usually at the tip of the villus, with capillary congestion
2	Extension of the subepithelial space with the moderate lifting of the epithelial layer
3	Massive epithelial lifting down the sides of villi
4	Denuded villi with lamina propria, dilated capillaries exposed, increased cellularity of the lamina propria
5	Digestion and disintegration of the lamina propria, hemorrhage and ulceration

Source: Elaborated by the authors.

### Statistical analysis

Data were analyzed using Statistical Package for the Social Sciences version 20 (IBM, Chicago, IL, United States of America). Normality was confirmed using the Shapiro-Wilk’s test. Parametric data were expressed as mean ± standard deviation (SD). Group comparisons were made using independent samples t-tests, and multiple comparisons (sham, I/R, I/R + PC) for histologic scores employed the Bonferroni post-hoc test. *P* < 0.05 was considered statistically significant.

## Results

Blood and tissue samples were biochemically analyzed. The results were evaluated statistically. Firstly, sham and control groups were compared to evaluate whether ischemia–reperfusion injury (IRI) occurred or not. The PC treatment group and control group were then compared.

No statistically significant result was found between the sham group and the IR group and between the IR group and the IR + PC group in terms of TAC values in plasma and tissue (*p* > 0.05). In terms of TOS and OSI values, there was a statistically significant decrease between sham group and IR group and between IR group and IR + PC group (*p* < 0.05, *p* < 0.05) (Table 2).

**Table 2:** Tissue and serum oxidative stress parameters of the subjects within groups.

	Sham	IR	IR + PC	*p*-value
Serum TAC (mmol Trolox equivalent/L	0.95 ± 0.17	0.93 ± 0.11	1.05 ± 0.25	> 0.05
Serum TOS (µmol H_2_O_2_ equivalent/L)	46.9 ± 16.7	80.8 ± 13.5	62.5 ± 18.9	< 0.05
Serum OSI	5 ± 1.7	8.8 ± 1.6	6.1 ± 2	< 0.05
Tissue TAC (mmol Trolox equivalent/L)	0,15 ± 0,06	0.15 ± 0.05	0.17 ± 0.04	> 0.05
Tissue TOS (mmol Trolox equivalent/L)	5.33 ± 1.35	10.03 ± 2	6.04 ± 2	< 0.05
Tissue OSI	4.03 ± 2.06	6,99 ± 2.23	3.97 ± 2.77	< 0.05

TAC: total antioxidant capacity; TOS: total oxidant status; OSI: oxidative stress index; IR: ischemia-reperfusion; PC: pinocembrin. Source: Elaborated by the authors.

**Figure 1:**
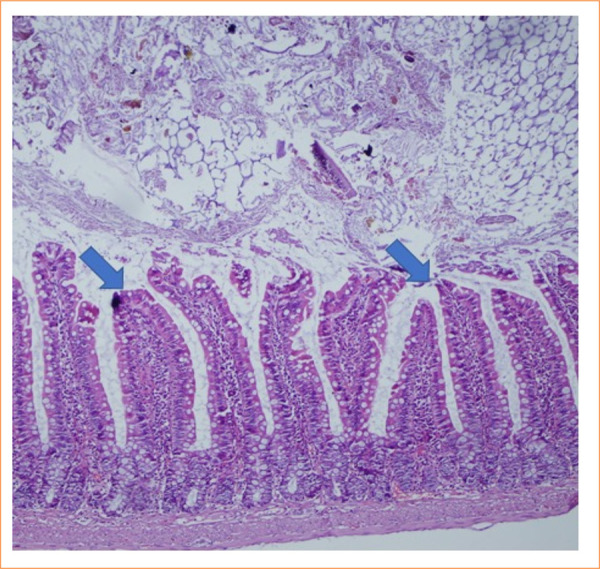
Histological characteristics of normal ileal tissue were observed (score = 0) (hematoxylin and eosin 200x).

Source: Elaborated by the authors.

**Figure 2:**
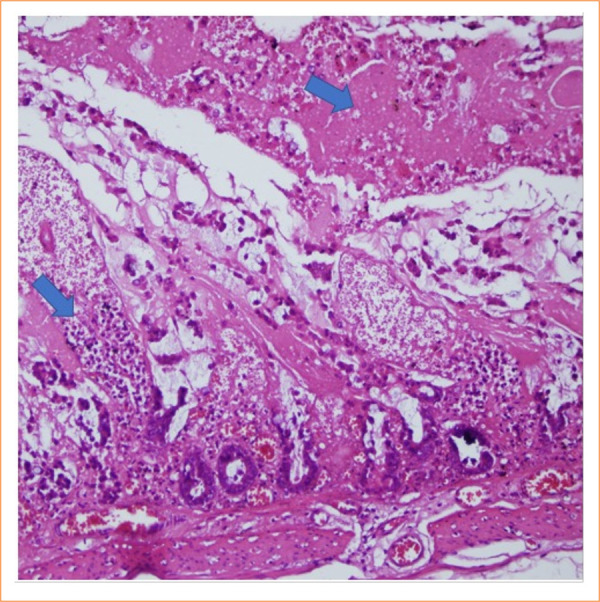
In the sample in the control group, disintegration, ulceration, and hemorrhage in the lamina propria were observed (score = 5) (hematoxylin and eosin 200x).

Source: Elaborated by the authors.

**Figure 3:**
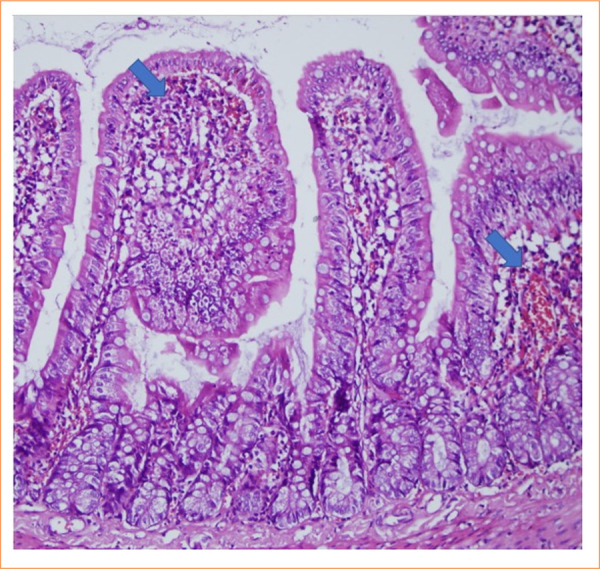
In the sample in the pinocembrin group, capillary congestion and edema were observed in the villi (score = 1) (hematoxylin and eosin 200x).

Source: Elaborated by the authors.

**Figure 4:**
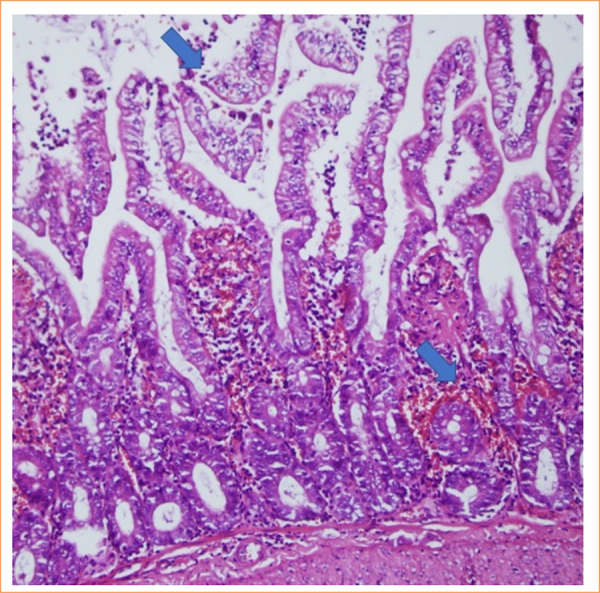
In the sample from the pinocembrin group, it was observed that the epithelial layer is separated from the lamina propria (score = 2) (hematoxylin and eosin 200x).

Source: Elaborated by the authors.

The intestinal mucosa of rats in the sham group was observed to be pathologically normal (Fig. 1). In the control group, extensive lamina propria disintegration, ulceration, and hemorrhage were observed pathologically (Fig. 2). As shown by histological staging, ischemic damage was significantly reduced in the PC-treated groups. Pathological examination in the PC group showed capillary congestion, edema, and separation of the epithelial layer from the lamina propria in the villi (Figs. 3 and 4). Statistically significant difference was found between the sham group and the IR group (*p* < 0.001). A significant difference was found between the IR group and the PC group (*p* = 0.009), but no significant difference was found between the PC group and the sham group (*p* = 0.15). Histological staging results are presented in detail in Table 3.

**Table 3:** Results of histological staging of ischemia reperfusion injury based on Chiu score.

	Mean ± SD	*p*-value
Sham	0 ± 0.00	*p* < 0.001*
IR group	2,6 ± 1,56	0.009*
PC Group	1 ± 1.05	0.15*

* Bonferroni test;

IR: ischemia-reperfusion; PC: pinocembrin; SD: standard deviation. Source: Elaborated by the authors.

## Discussion

In the present study, PC administration significantly reduced oxidative damage (TOS, OSI) and histological injury in a rat model of intestinal I/R. By contrast, TAC levels did not differ among the groups.

Intestinal I/R is the restoration of blood flow to the intestine following inadequate blood supply, which can occur in various clinical scenarios caused by diseases such as acute mesenteric embolism or thrombus, strangulated hernias, severe burns or traumatic or septic shock. Tissue hypoxia resulting from intestinal ischemia and disturbances in cellular energy metabolism due to nutrient deficiency can lead to a variety of pathophysiological mechanisms, including the formation of free oxygen radicals as a result of impaired mitochondrial function^19^^–^ 22. As a result of these changes, intracellular stress increases, resulting in cell death. After reperfusion is achieved, the ischemic area is re-circulated. This allows cells to regain access to oxygen and nutrients. However, this process can be also detrimental, as the reintroduction of blood to ischemic tissue during reperfusion can lead to reperfusion injury and eventually cell death^23^. Unfortunately, there is still no effective treatment for intestinal I/R injury, leading to a high mortality rate. According to statistics, 26% of patients do not live more than one year^21^.

PC has shown potent antioxidant properties through the induction of endogenous antioxidant capacity by various mechanisms^24^. In addition, it has anti-inflammatory activities shown in various disease models and different organs.

Studies with PC have shown that it inhibits the production of inflammatory cytokines, including tumor necrosis factor (TNF)-α, interleukin (IL)-1β and IL-6, and increases the level of anti-inflammatory IL-10 in activated macrophages^25^. In rats with ulcerative colitis, low doses of PC administered orally for one week decreased the expression of TNF-α, IL-1β, and IL-6 and increased the level of transforming growth factor (TGF)-β, which is essential for healing of the intestinal mucosa.

This study demonstrated a protective effect of PC against ulcerative colitis^26^. However, another study in rats using higher doses also demonstrated the therapeutic effect of PC and showed that its anti-inflammatory effects in ulcerative colitis are probably due to inhibition of the TLR4/NF-κB pathway^15^. PC also showed beneficial effects in neuroinflammation in different models^13^. In a rat ischemic stroke model, it inhibited the stimulation of microglia and astrocytes and decreased the expression of TNF-α, IL-1β, ıntercellular adhesion molecule (ICAM)-1, vascular cell adhesion molecule (VCAM)-1, inducible NO synthase (iNOS), and aquaporin-4^27^. Although direct experimental data on PC’s impact in intestinal IR remain limited, its protective action in related gastrointestinal pathologies (*e.g.*, colitis, sepsis-related gut injury) strongly supports our results.

In our study, we measured total oxidant and antioxidative capacities simultaneously to evaluate oxidative stress more accurately. We investigated both TAC and TOS using the measurement methods developed by Erel^16^^,^ 17. With OSI, we evaluated oxidative stress using both oxidative and antioxidative parameters. The determination of TAC is a very useful method in determining the protective capacity of the organism against free oxygen radicals. Similarly, TOS is used to determine the oxidative power of free radicals in the organism^28^. TOS measurement provides a sensitive measure of lipid peroxidation and oxidative stress. However, these markers have mostly been measured in serum. In our study, unlike other studies, we measured TAC, TOS, and OSI levels in both plasma and tissue. In our study, there was a decrease in TOS and OSI values at the tissue and level, possibly suggesting that PC chiefly mitigates oxidative burden rather than amplifying the global antioxidant capacity in this model. These data align with research indicating that PC exerts powerful anti-inflammatory and ROS-scavenging effects in various tissues^11^^,^ 12.

Several limitations warrant attention. First, only one dose (5 mg/kg) of PC and a single I/R interval (60 min ischemia + 60 min reperfusion) were used. Dose-response and time-course studies might clarify whether longer reperfusion intervals or higher doses would yield greater TAC changes. Second, while animals were maintained under standard laboratory conditions, they were not fully SPF, which may influence inflammatory responses. Nonetheless, the observed reductions in tissue TOS, OSI, and histopathological damage strongly indicate a protective effect of PC.

Future research should explore optimal dosing and timing regimens and investigate the molecular mechanisms underlying PC-mediated protection–specifically its potential modulation of intracellular signaling pathways such as TLR4/NF-κB or the Nrf2/HO-1 axis^24^. Clinical studies are also needed to validate the safety and efficacy of PC in human intestinal IR scenarios.

## Conclusion

PC significantly attenuated intestinal I/IR in rats, as evidenced by reduced oxidative stress and milder histopathological damage. These findings highlight PC as a promising candidate for further research into intestinal I/R therapeutics. However, additional mechanistic studies and dose-optimization protocols are essential to fully elucidate the clinical potential of this natural flavonoid.

## Data Availability

The datasets used and/or analyzed during the current study are available from the corresponding author upon reasonable request.

## References

[1] Bala M, Catena F, Kashuk J, De Simone, Gomes CA, Weber D, Sartelli M, Coccolini F, Kluger Y, Abu-Zidan FM, Picetti E, Ansaloni L, Augustin G, Biffl WL, Ceresoli M, Chiara O, Chiarugi M, Coimbra R, Cui Y, Damaskos D, Di Saverio, Galante JM, Khokha V, Kirkpatrick AW, Inaba K, Leppäniemi A, Litvin A, Peitzman AB, Shelat VG, Sugrue M, Tolonen M, Rizoli S, Sall I, Beka SG, Di Carlo, Ten Broek, Mircea C, Tebala G, Pisano M, van Goor, Maier RV, Jeekel H, Civil I, Hecker A, Tan E, Soreide K, Lee MJ, Wani I, Bonavina L, Malangoni MA, Koike K, Velmahos GC, Fraga GP, Fette A, de’Angelis N, Balogh ZJ, Scalea TM, Sganga G, Kelly MD, Khan J, Stahel PF, Moore EE (2022). Acute mesenteric ischemia: updated guidelines of the World Society of Emergency Surgery. World J Emerg Surg.

[2] Carden DL, Granger DN (2000). Pathophysiology of ischaemia–reperfusion injury. Pathophysiology of ischaemia–reperfusion injury.

[3] Vollmar B, Menger MD (2011). Intestinal ischemia/reperfusion: microcirculatory pathology and functional consequences. Langenbecks Arch Surg.

[4] Zhang M, Liu Q, Meng H, Duan H, Liu X, Wu J, Gao F, Wang S, Tan R, Yuan J (2024). Ischemia-reperfusion injury: molecular mechanisms and therapeutic targets. Signal Transduct Target Ther.

[5] Eltzschig HK, Eckle T (2011). Ischemia and reperfusion--from mechanism to translation. Nat Med.

[6] Bosi A, Banfi D, Bistoletti M, Catizzone LM, Chiaravalli AM, Moretto P, Moro E, Karousou E, Viola M, Giron MC, Crema F, Rossetti C, Binelli G, Passi A, Vigetti D, Giaroni C, Baj A (2022). Hyaluronan regulates neuronal and immune function in the rat small intestine and colonic microbiota after ischemic/reperfusion injury. Cells.

[7] Fan S, Xu Y, Li K, Li B, Diao Y (2022). Ellagic acid alleviates mice intestinal ischemia-reperfusion injury: a study based on transcriptomics combined with functional experiments. Chem Biodivers.

[8] Liao S, Luo J, Kadier T, Ding K, Chen R, Meng Q (2022). Mitochondrial DNA release contributes to intestinal ischemia/reperfusion injury. Front Pharmacol.

[9] Gu J, Huang H, Liu C, Jiang B, Li M, Liu L, Zhang S (2021). Pinocembrin inhibited cardiomyocyte pyroptosis against doxorubicin-induced cardiac dysfunction via regulating Nrf2/Sirt3 signaling pathway. Int Immunopharmacol.

[10] Rasul A, Millimouno FM, Ali Eltayb W, Ali M, Li J, Li X (2013). Pinocembrin: a novel natural compound with versatile pharmacological and biological activities. Biomed Res Int.

[11] Hu L, Wu C, Zhang Z, Liu M, Maruthi Prasad E, Chen Y, Wang K (2019). Pinocembrin protects against dextran sulfate sodium-induced rats colitis by ameliorating inflammation, improving barrier function and modulating gut microbiota. Front Physiol.

[12] Zhou Y, Wang D, Yan W (2023). Treatment effects of natural products on ınflammatory bowel disease ın vivo and their mechanisms: based on animal experiments. Nutrients.

[13] Wang P, Pan L, Liu Q, Huang Y, Tang Y, Lin B, Liao Y, Luo H, Meng X (2024). Pinocembrin activation of DPP9 inhibits NLRP1 inflammasome activation to alleviate cerebral ischemia/reperfusion-induced lung and intestinal injury. Immunol Res.

[14] Hu L, Wu C, Zhang Z, Liu M, Maruthi Prasad E, Chen Y, Wang K (2019). Pinocembrin Protects Against Dextran Sulfate Sodium-Induced Rats Colitis by Ameliorating Inflammation, Improving Barrier Function and Modulating Gut Microbiota. Front Physiol.

[15] Elbatreek MH, Mahdi I, Ouchari W, Mahmoud MF, Sobeh M (2023). Current advances on the therapeutic potential of pinocembrin: An updated review. Biomed Pharmacother.

[16] Erel O (2004). A novel automated direct measurement method for total antioxidant capacity using a new generation, more stable ABTS radical cation. Clin Biochem.

[17] Erel O (2005). A new automated colorimetric method for measuring total oxidant status. Clin Biochem.

[18] Chiu CJ, McArdle AH, Brown R, Scott HJ, Gurd FN (1970). Intestinal mucosal lesion in low-flow states. I. A morphological, hemodynamic, and metabolic reappraisal. Arch Surg.

[19] Tilsed JV, Casamassima A, Kurihara H, Mariani D, Martinez I, Pereira J, Ponchietti L, Shamiyeh A, Al-Ayoubi F, Barco LA, Ceolin M, D’Almeida AJ, Hilario S, Olavarria AL, Ozmen MM, Pinheiro LF, Poeze M, Triantos G, Fuentes FT, Sierra SU, Soreide K, Yanar H (2016). ESTES guidelines: acute mesenteric ischaemia. Eur J Trauma Emerg Surg.

[20] Stone JR, Wilkins LR (2015). Acute mesenteric ischemia. Tech Vasc Interv Radiol.

[21] Wang J, Zhang W, Wu G (2021). Intestinal ischemic reperfusion injury: Recommended rats model and comprehensive review for protective strategies. Biomed Pharmacother.

[22] Xu S, He Y, Lin L, Chen P, Chen M, Zhang S (2021). The emerging role of ferroptosis in intestinal disease. Cell Death Dis.

[23] Hu J, Deng F, Zhao B, Lin Z, Sun Q, Yang X, Wu M, Qiu S, Chen Y, Yan Z, Luo S, Zhao J, Liu W, Li C, Liu KX (2022). Lactobacillus murinus alleviate intestinal ischemia/reperfusion injury through promoting the release of interleukin-10 from M2 macrophages via Toll-like receptor 2 signaling. Microbiome.

[24] Habtemariam S (2019). The Nrf2/HO-1 axis as targets for flavanones: neuroprotection by pinocembrin, naringenin, and eriodictyol. Oxid Med Cell Longev.

[25] Soromou LW, Chu X, Jiang L, Wei M, Huo M, Chen N, Guan S, Yang X, Chen C, Feng H, Deng X (2012). In vitro and in vivo protection provided by pinocembrin against lipopolysaccharide-induced inflammatory responses. Int Immunopharmacol.

[26] Yue B, Ren J, Yu Z, Luo X, Ren Y, Zhang J, Mani S, Wang Z, Dou W (2020). Pinocembrin alleviates ulcerative colitis in mice via regulating gut microbiota, suppressing TLR4/MD2/NF-κB pathway and promoting intestinal barrier. Biosci Rep.

[27] Gao M, Zhu SY, Tan CB, Xu B, Zhang WC, Du GH (2010). Pinocembrin protects the neurovascular unit by reducing inflammation and extracellular proteolysis in MCAO rats. J Asian Nat Prod Res.

[28] Rabus M, Demirbağ R, Sezen Y, Konukoğlu O, Yildiz A, Erel O, Zeybek R, Yakut C (2008). Plasma and tissue oxidative stress index in patients with rheumatic and degenerative heart valve disease. Turk Kardiyol Dern Ars.

